# *P21 *Ser31Arg and *FGFR2* rs2981582 Polymorphisms as Risk Factors for Early Onset of Breast Cancer in Yogyakarta, Indonesia

**DOI:** 10.31557/APJCP.2019.20.11.3305

**Published:** 2019

**Authors:** Dewajani Purnomosari, Clarista Raharjo, Alvin Santoso Kalim, Rahma Herviastuti, Markus Yushan, Rangga Athilah Fajar, Karina Kazia Harris, Artanto Wahyono

**Affiliations:** 1 *Department of Histology and Cell Biology, *; 3 *Department of Surgery, Faculty of Medicine, *; 2 *Undergraduate Programme, Public Health and Nursing, Universitas Gadjah Mada, Jl. Farmako, Sekip Utara, Yogyakarta, Indonesia. *

**Keywords:** Age of onset, breast carcinoma, FGFR2, P21, single nucleotide polymorphism

## Abstract

**Objectives:**

Breast cancer tend to be more progressive with poorer prognosis in younger patients than those at an older age. Single Nucleotide Polymorphisms (SNPs) of *P53* Pro72Arg, *MDM2* SNP309, *P21* Ser31Arg, *ER* SNP594, *HER2 *Ile655Val, and *FGFR2* rs2981582 have drawn attention as genetic factors associated with cancer risk. However, there were contradictory results involving different races and their association is still unknown in Indonesian populations. This study was performed to examine the proportion of these six genes polymorphisms and their associations with age of onset of breast cancer patients in Yogyakarta, Indonesia.

**Methods:**

Biorepository DNA from 199 patients registered at Dr. Sardjito Hospital Yogyakarta from 2006-2013 were tested for polymorphisms using the PCR-RFLP method. Samples were taken from two age groups; early-onset (<40 years) and late-onset of breast cancer (>55 years). Chi-square tests with odds ratio were used for data analysis.

**Results:**

The mean age of the early-onset group was 36**±**4.2 years, while the late-onset group was 62**±**6.9 years. AA genotype and A allele of *P21* and TT genotypes and T allele of *FGFR2* were significantly more frequent and were associated with an increased risk of early-onset of breast cancer (95%CI: 2.54 and 1.59; 2.63 and 1.64, respectively).

**Conclusions:**

Our study indicates that the A allele of *P21* and the allele T of *FGFR2* may be associated with an increased risk of early-onset of breast cancer in Yogyakarta, Indonesia. Further analysis is needed to confirm the findings.

## Introduction

Globally, breast cancer (BC) ranks highest and is the most prevalent carcinoma among women in Indonesia, accounting for 48.998 new cases (30.5%) with 5 years prevalence of 171.005 (41.7%) (Ferlay, 2013). Early onset BC occurs in patients aged 40 years or younger and its incidence varies among races (Anders et al., 2009). Early onset ofBC tends to occur with hormone receptor negativity in larger and more aggressive, high grade tumors with hormone receptor negativity, and *HER2 *overexpression (Assi et al., 2013), with greater risk of contralateral breast cancer in 15 years (Metcalfe et al., 2011). 

In the United States, only 6.8% of BC patients are below 40 years (Anders et al., 2009), whereas in Asia its incidence rate could reaches 9.5-11% (Han et al., 2004). Early onset cases in Dr. Sardjito Hospital Yogyakarta between the years 2012–2015 showed incidence rates ranging from 12.8 – 19.7% (unpublished data). 

One of the major risk factors for early onset BC is a history of BC in the family, which involves inherited genetic mutations, in particular the major susceptibility genes *BRCA1* and *BRCA2*. However, *BRCA1* and *BRCA2* have been shown to account for up to only 15% of familial early onset BC cases (Stratton and Rahman, 2008). One study in China showed that among early-onset BC without a family history of ovarian or BC, these genes were responsible for about 3% and 0%, respectively (Chen et al., 2009). The same type of study in Indonesia showed that *BRCA1* and *BRCA2* were responsible for 8.3% of the cases (Purnomosari et al., 2007). , accounts for only a small percentage of early-onset non-familial BC, indicating other low penetrance susceptibility genes could exist and play a role in tumorigenesis. 

Genetic mutations occur in about 1% of the population and the most common type of germline variations are single nucleotide polymorphisms (SNPs) (Taylor et al., 2001). While SNP usually have little effect individually, polygenic models indicate combinations of genetic mutation, may together contribute to higher risk of cancer (Pharoah et al., 2002). A number of SNPs of several genes have been associated with susceptibility to early onset of BC, such as *HER2 *Ile655Val (Montgomery et al., 2003), *MDM2* SNP309 and *p53* Arg72Pro (Sun et al., 2009), ER1 594G to A (Yu et al., 2006) and *p21* Ser31Arg (Ma et al., 2006; Qiu et al., 2010). Recent genome-wide association studies have identified genetic variations that may play a role as risk factors for breast cancer in populations of diverse ethnicity (Easton et al., 2007). SNP rs2981582 which lies in intron 2 of *FGFR2* gene is the most significantly associated SNP among eleven SNPs that have been linked with BC (Easton et al., 2007). Further studies also proved the role of *FGFR2* rs2981582 as a susceptible gene for early onset of BC (Raskin et al., 2008; Siddiqui et al., 2014), even though like other low penetrance genetic variant studies, the results are not consistent among populations. Thus, the present study aimed to examine the genotypes of *p53* Arg72Pro, *MDM2* SNP309 T to G, *p21* Ser31Arg, *HER2 *Ile655Val, ER SNP594 G to A, and *FGFR2* rs2981582 and their allele frequencies in a clinical group of early onset and late onset BC patients and to test for any correlations between certain genotypes or alleles with the risk of developing early onset BC in Yogyakarta, Indonesia.

## Introduction

*Study population and sample collection*

This research was a hospital-based case control study that included 98 early-onset BC cases (< 40 years old at diagnosis) and 101 late-onset cases (> 55 years old) as the controls. These cases were pathologically diagnosed and recruited between the year 2006 – 2013 at the Dr. Sardjito Hospital Yogyakarta Province Indonesia. The study received approval from the Medical and Health Research Ethics Committee of Faculty of Medicine Universitas Gadjah Mada (KE/FK/779/EC and KE/FK/570/EC/2015).

*DNA extraction and genotyping*

Spectrophotometric measurement of the absorbance ratio 260/280 nm were used to determine DNA purity and concentration. Genotyping of the 6 genes was performed using Polymerase chain reaction-restriction fragment length polymorphisms (PCR-RFLP) assay. Details of selected SNPs, primer sequences used for PCR, sizes of PCR products and restriction endonuclease, (New England Biolabs, Ipswich, Massachusetts, United States) used for digestion are described (Table 1). Restriction digested fragments from all of the SNPs were analyzed with 3% agarose gel electrophoresis (except for *FGFR2*, 4% of agarose).

*Statistical analyses*

Data analysis used SPSS-21 software (IBM Corp., Chicago). The Hardy-Weinberg equilibrium was first applied to both case and control groups, separately for each variant before association analyses. Significance level (α) used was 5%. To calculate odds ratios (ORs), unconditional logistic regression model were used with 95% confidence interval (CI) as estimates of relative risk (RR) for the single locus alleles and genotypes. 

**Table 1:** Primers and Restriction Enzyme Used for Genotyping 6 Genes

Gene	SNP	rs number		primer	PCR product	enzyme
p53	Pro72Arg	rs1042522	F	5'- TTG CCG TCC CAA GCA ATG GAT GA -3'	199bp	Bstu1
			R	5'- TCT GGG AAG GGA CAG AAG ATG A -3'		
MDM2	309 T>G	rs2279744	F	5'- GAT TTC GGA CGG CTC TCG CGG C-3'	121bp	PstI
			R	5'- CAT CCG GAC CTC CCG CGC TG-3'		
HER2	Ile655Val	rs1136201	F	5'- AGA GAG CCA GCC CTC TGA CGT CCA T- 3'	148bp	BsmAI
			R	5'- TCC GTT TCC TGC AGC AGT CTC CGC A -3'		
ER1	594 A>G	rs2228480	F	5'- GAG GAG ACG GAC CAA AGC CAC -3'	227bp	BtgI
			R	5'- GCC ATT GGT GTT GGA TGC ATG C -3'		
p21	Ser31Arg	rs1801270	F	5'- ATA GTG TCT AAT CTC CGC CG -3'	245bp	Blp1
			R	5'- AAG TCA CCC TCC AGT GGT GT -3'		
FGFR2	-	rs2981582	F	5'- CCC TTT GGA GAC AAC GTG AGC C- 3'	176bp	HinP1I
			R	5'- CAG GCA CCA GGT GGA CTC TGC-3'		

**Table 2:** Genotype and Allelic Frequencies of P53 Pro72Arg, MDM2 SNP309, P21 Ser31Arg, ER SNP594, HER2 Ile655Val, and FGFR2 rs2981582 in Breast Cancer Patients with Different Age of Onset

Polymophisms	Genotype/Allele	Cases (<40) n=98	Controls (>=55) n=101	p-value	OR	95% CI
HER2 Ile133Val	I/I	73 (74.5%)	74 (73.3%)	0.614	ref	ref
	I/V	21 (21.4%)	25 (24.8%)		0.852	0.42-1.74
	V/V	4 (4.1%)	2 (2.0%)		2,027	0.31-16.51
	I	167 (85.2%)	173 (85.6%)	0.901	1,036	0.57-1.87
	V	29 (14.8%)	29 (14.3%)			
ER1 594A>G	A/A	5 (5.1 %)	5 (5%)	0.52	ref	ref
	A/G	35 (35.7%)	44 (43.6%)		0.713	0.38-1.33
	G/G	58 (59.2%)	52 (51.5%)		0.897	0.21-3.84
	A	45 (23%)	54 (26.7%)	0.384	0.817	0.51-1.32
	G	151 (77%)	148 (73.3%)			
p53 Arg72Pro	A/A	33 (33.7%)	25 (24.8%)	0.183	ref	ref
	A/P	47 (48%)	50 (49.5%)		0.637	0.31-1.29
	P/P	18 (18.4%)	26 (25.7%)		0.489	0.20-1.17
	A	114 (58.2%)	99 (49.0%)	0.067	0.691	0.46-1.05
	P	82 (41.8%)	103(51.0%)			
MDM2 SNP309	T/T	20 (20.5%)	23 (22.8%)	0.644	ref	ref
	T/G	54 (55.1%)	49 (48.5%)		0.789	0.39-1.61
	G/G	24 (24.5%)	29 (28.7%)		1.051	0.47-2.36
	T	94 (48.0%)	95 (47.0%)	0.853	1.038	0.7-1.54
	G	102 (52.0%)	107 (53.0%)			
p21 Ser31Arg	C/C	21 (21.4%)	30 (29.7%)	0.047*	ref	ref
	C/A	45(45.9%)	53(52.5%)		1.213	0.61-2.41
	A/A	32(32.7%)	18 (17.8%)		2.54	1.14-5.67
	C	87 (44.4%)	113 (56%)	0.021*	1.591	1.07-2.36
	A	109 (55.6%)	89 (44%)			
FGFR2 rs2981582	C/C	26(26.5%)	37 (36.6%)	0.091	ref	ref
	C/T	45 (45.9%)	48 (47.5%)		1.334	0.70-2.55
	T/T	27(27.5%)	16 (15.8%)		2.401	1.08-5.32
	C	97 (49.5%)	122 (60.4%)	0.028*	1.556	1.05-2.32
	T	99 (50.5%)	80 (39.6%)			

*, p<0.05; OR, odds ratio; CI, confidence interval

## Results

In this hospital-based case and control study, we analyzed 6 genes’ polymorphisms in 98 early-onset BC patients and 101 late-onset BC patients as the controls. The mean age at diagnosis for early-onset group was 36**±**4.2 years, while the late-onset group was 62**±**6.9 years. The Hardy-Weinberg equilibrium analysis showed no significant deviations between early and late-onset BC patients (p>0.05; data not shown).

Genotype distribution of each evaluated gene can be found in Table 2. Four genes showed high frequency in heterozygote genotypes, i.e. Arg/Pro of *p53*, T/G of *MDM2*, A/C of *p21* and C/T of *FGFR2* with proportion of 48%, 55.1%, 45.9% and 45.9%, respectively. There was no different proportion between early and late onset cases. On the other hand, genotype frequencies of *HER2 *and ER1 were different from the other 4 genes, as the most frequent genotype for *HER2 *was the homozygote wildtype (Ile/Ile), while for ER1 it was the homozygote mutant (G/G). 

Genotype C/C of *P21* was mostly found in late onset compared to early onset cases (29.7% vs 21.4%), while frequency of genotype A/A was higher in early onset compared to late onset (32.7% vs 17. 8%). There were significant differences in frequency of p21 A/A and allele A found through Chi square tests in the younger group compared to older cases (p=0.047 and p=0.021, respectively). Similar circumstance was found in *FGFR2* genes, where frequency of C/C genotype was higher in late onset compared to early onset (36.6% vs 26.5%), while T/T genotype was higher in early onset compared to late onset cases (27.5% vs 16.8%), but the difference was not statistically significant. Allele T was more frequently found in early onset cases compare to late onset (50.5% vs 39.6%), and the difference was statistically significant (p=0.028). 

Logistic regression models comparing with Ser/Ser (C/C genotype) of *P21*, found those with Ser/Arg (C/A) and Arg/Arg (A/A) had OR of 1.213 (95% CI: 0.612-2.405) and 2.540 (95% CI: 1.138–5.668), respectively, suggesting that Ser31Arg polymorphisms are correlated with the increased risk of early onset BC. Similarly, T/T genotype and T allele of *FGFR2* rs2981582 were found to increase the risk of early onset BC with OR of 2.40 (95% CI: 1.08–5.32) and 1.56 (95% CI: 1.05–2.32) respectively. 

## Discussion

Poor prognosis and low survival are linked to early onset of BC and accordingly, preventive measures by recognizing the risk factors are very much needed. Genetic variations in genes that regulate crucial processes such as cell apoptosis, proliferation and DNA repair are potential candidates for breast cancer risk factors. Recent research that was conducted in many different countries shows different outcomes among different populations.

In this study we evaluated 6 genes thathave been linked with susceptibility to early onset of BC, only two of them, *P21* and *FGFR2* were associated as risk factors in an Indonesian population.

*P21 Se31Arg polymorphism*

Results of the present study showed that AA genotype and A allele of *P21* were significantly more frequently found in early onset cancer cases and had a 2.540 increased risk of early-onset BC (95% CI: 1.138–5.668). *P21* (also identified as CDKN1A), which encodes *p21*WAF1/CIP1, a cyclin-dependent kinase inhibitor, is considered to significantly influence cell cycle control (Gartel and Radhakrishnan, 2005). One polymorphism of *P21* that has received much attention is Ser31Arg (rs1801270), which substitutes A for C in the third base of codon 31 of *P21* results in a serine to arginine amino acid substitution in the DNA-binding zinc finger motif of the protein (Mousses et al., 1995). Thus, it is very likely this polymorphism is also involved in cancer development (Lukas et al., 1997). 

Several results support the role of *P21* Ser31Arg polymorphism in BC risk (Keshava et al., 2002; Ma et al., 2006), but the results are inconclusive, possibly due to small effect of the polymorphism on BC risk and the relatively small number of samples used in the studies. 

There have been several studies of *P21* Ser31Arg polymorphism that were done to determine the risk of BC incidence. One meta-analysis study by Qiu and colleagues (Qiu et al., 2010) reported that the genotype C/C significantly increased the risk of breast cancer by 1.496 times (95% CI:1.164-1.924) for Caucasians. However, for the Asian population, there was no statistically significant increase in risk (OR 0.896; 95% CI:0.424-1.893). 

*FGFR2 rs2981582 polymorphism*

*FGFR2* is a member of the FGFR family that contributes to cell growth, malignancy, motility, and angiogenesis. Various meta-analysis studies have been conducted and reported that SNP *FGFR2* rs2981582 TT is linked to an increased risk of BC, but until now the exact mechanism of carcinogenesis linking SNP *FGFR2* rs2981582 TT to BC, especially early breast cancer onset has not been found (Zhang et al., 2010; Peng et al., 2011). In this study, frequency of C/C genotype was higher in patient with late onset compared to early onset (36.6% vs 26.5%) while T/T genotype was higher in younger cases compared to the older patients (27.5% vs 16.8%), but the result was not statistically significant. Interestingly, proportion of allele T was significantly different in early onset cases. 

SNP *FGFR2* rs2981582 intron 2 alters the binding of two transcription factors, Oct-1 / Runx2 and C / EBpb, resulting in increased expression of the *FGFR2* gene in cell lines and mammary tissue (Jia et al., 2010). This overexpression is confirmed by the study of Rebbeck (Rebbeck et al., 2009) that reported *FGFR2* over expression occurs in 10-15% of breast tumors, but *FGFR2* expression is lower in normal breast tissue in someone who has T allele (Sun et al., 2010). In addition, the in vitro evidence also shows that the mechanism of carcinogenesis of the SNP is more directed toward the anti-apoptosis effect than the mitogenic effects which are partly mediated through the down-regulation of FOXO protein synthesis. Amplification of the *FGFR2* gene due to SNP results in an increase in intracellular screening which affects the decrease of FOXO protein. FOXO proteins are involved in apoptotic induction, DNA damage repair, ROS detoxification, and down-regulation of ER-alpha and ER-beta transcription activities. Therefore, decreased protein synthesis of FOXO will decrease apoptosis, ROS detoxification function, DNA repair, and down-regulation of ER transcription activity (Murillo-Zamora et al., 2013). 

All of the above findings significantly support our results that show *FGFR2* gene polymorphism rs2981582 TT can be linked to increased risk of early onset BC in Yogyakarta, Indonesia. This finding is consistent with a study conducted by Fu et al., (2012) which stated that TT and T allele genotypes in *FGFR2* polymorphism rs2981582 increase the risk of early-onset BC in South Chinese Han populations with ORs of 3.062 (95% CI: 1.229-7.629) and 1.606 (95% CI: 1.084-2.380), respectively. But in the study, the controls used were healthy women, as well as the age-appropriate early-onset BC limit was <35 years. There is no similar study that discusses the relationship of early onset of breast cancer and *FGFR2* polymorphism in other populations, but a study by Liang (Liang et al., 2008) found that there is a strong association of TT mutant genotypes and T alleles in *FGFR2* polymorphism rs2981582 as risk factors of occurrence of BC in premenopausal women. 

Our study indicates that the A allele of *P21* and the allele T of *FGFR2* may be linked to increased risk of early-onset BC in Yogyakarta, Indonesia. In order to confirm the findings, further analyses are needed. 

*Funding statement*

This study received grant support from Dana Masyarakat Faculty of Medicine Universitas Gadjah Mada 2015 and 2016.

*Presentation statement*

The manuscript has been presented as poster presentation in 9th General Assembly and International Conference 2018. Asian Pacific Organization for Cancer Prevention, Jeju Island, South Korea, 19-20 April 2018
